# Supplementary material for: A hexagon in Saturn’s northern stratosphere surrounding the emerging summertime polar vortex
Source: Nat Commun. 2018 Sep 3;9:3564. doi: 10.1038/s41467-018-06017-3 (PMC6120878; doi:10.1038/s41467-018-06017-3)
Supplement: Supplementary file 1 — Supplementary Information [file 41467_2018_6017_MOESM1_ESM.pdf]

Supplementary Material for Fletcher et al. A Hexagon in Saturn's Northern Stratosphere Surrounding the Emerging Summertime Polar Vortex

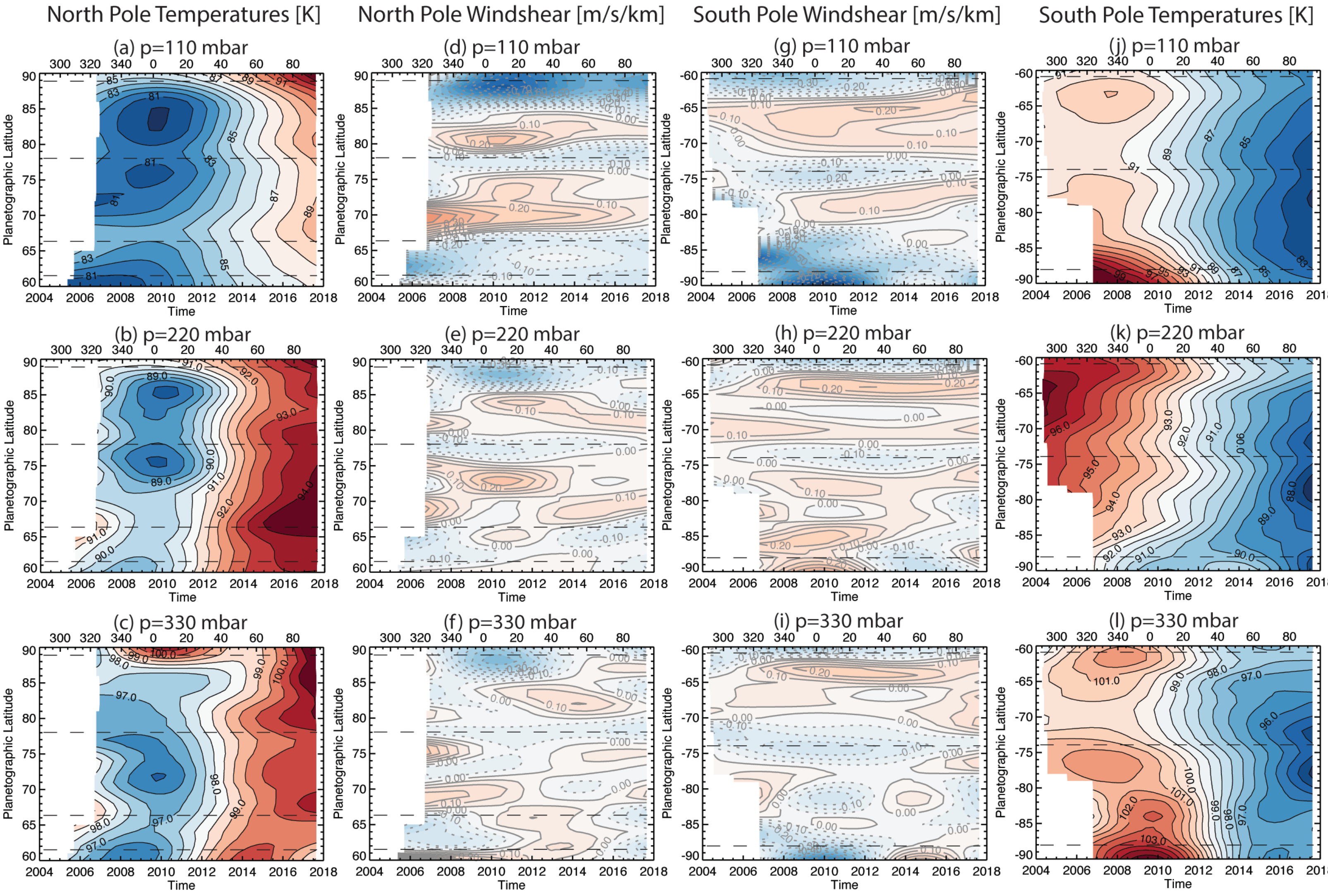

Figure 1: **Supplementary Fig. 1: North and South polar tropospheric temperature gradients as a function of time throughout the whole Cassini mission, 2004-2017**. We display north polar temperatures (panels a-c), north polar windshears (panels d-f), south polar windshears (panels g-i) and south polar temperatures (panels j-l) at three different pressure levels (110, 220 and 330 mbar). These were derived from averages of low-resolution CIRS spectra on a monthly temporal grid, and interpolated using tensioned splines[1] to reconstruct a smoothed temperature field. Horizontal dashed lines signify the peak of eastward zonal jets in the troposphere[2]. The data are displayed as a function of time (years), but a second horizontal axis provides the planetocentric solar longitude (L_s) in degrees.

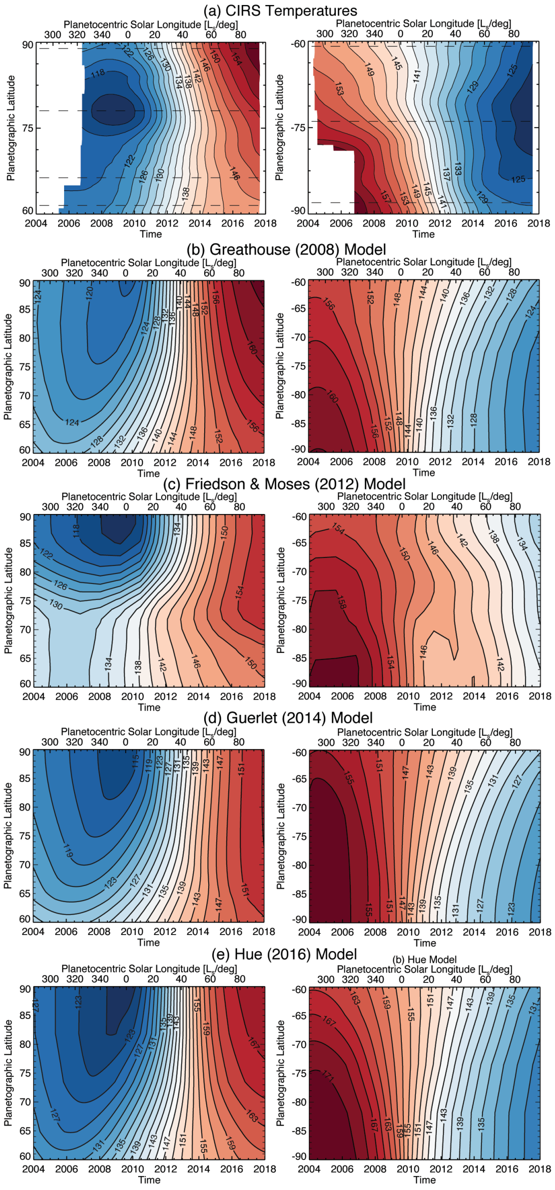

Figure 2: **Supplementary Fig. 2: Comparison of the 1-mbar temperature field to the predictions of radiative models.** We compare (a) the CIRS low-resolution measurements to predictions of (b) the radiative model of Greathouse et al.[3]; (c) the radiative-dynamical model of Friedson and Moses[4]; (d) the radiative-convective model of Guerlet et al.[5]; and (e) the combined radiative-photochemical model of Hue et al.[6]. Results for the north pole are shown on the left, and the south pole are shown on the right. Although the predicted temperatures are close to those observed, none of the models include the warming of the North Pole, and none of them predict the appearance of the strong $\partial T/\partial y$ gradients associated with the polar vortices.

Table 1: Supplementary Table 1: Sources of spectroscopic linedata and foreign broadening assumptions. Exponents for temperature dependence T^n given in the final column.

**Gas**	**Line Intensities**	**Broadening Half Width**	**Temperature Dependence**
CH_4, CH_3D	Brown et al.[7]	H_2 broadened using a half-width of 0.059 cm^{-1}atm^{-1} at 296 K	$n = 0.44$ Margolis et al.[8]
C_2H_6	Vander-Auwera et al.[9]	0.11 cm^{-1}atm^{-1} at 296 K Blass et al.[10]	$n = 0.94$ Halsey et al.[11]
C_2H_2	GEISA'03[12]	Fits to data in Varanasi et al.[13]	Varanasi et al.[13]
PH_3	Kleiner et al.[14]	Broadened by both H_2 and He using $\gamma_{H_2} = 0.1078 - 0.0014J$ cm^{-1}atm^{-1} and $\gamma_{He} = 0.0618 - 0.0012J$ cm^{-1}atm^{-1} Levy et al.[15], Bouanich et al.[16]	$n = 0.702 - 0.01J$ (J is the rotational quantum number) Salem et al.[17]
NH_3	Kleiner et al.[14]	Empirical model of Brown et al.[18]	Brown et al.[18]
C_2H_4	GEISA'03[12]	Polynomial fits to data from Bouanich et al.[19,20]	$n = 0.73$, Bouanich et al.[20]
C_3H_4	GEISA'09[21]	0.075 cm^{-1}atm^{-1} for all lines	$n = 0.50$ assumed
C_4H_2	GEISA'09[21]	0.1 cm^{-1}atm^{-1} for all lines	$n = 0.75$ assumed
C_3H_8	GEISA'09[21]	0.08 cm^{-1}atm^{-1} for all lines	$n = 0.75$ assumed.
C_6H_6	GEISA'09[21]	Linear fit to N_2-broadening of Waschull et al.[22]	$n = 0.75$ assumed

Supplementary References

1. Teanby, N. Constrained Smoothing of Noisy Data Using Splines in Tension. *Mathematical Geology* **39**, 419–434 (2007).

2. Antuñano, A., Río-Gaztelurrutia, T., Sánchez-Lavega, A. & Hueso, R. Dynamics of Saturn's polar regions. *Journal of Geophysical Research (Planets)* **120**, 155–176 (2015).

3. Greathouse, T. K. *et al.* A General Radiative Seasonal Climate Model Applied to Saturn, Uranus, and Neptune. *AGU Fall Meeting Abstracts* P21B–06 (2008).

4. Friedson, A. J. & Moses, J. I. General circulation and transport in Saturn's upper troposphere and stratosphere. *Icarus* **218**, 861–875 (2012).

5. Guerlet, S. *et al.* Global climate modeling of Saturn's atmosphere. Part I: Evaluation of the radiative transfer model. *Icarus* **238**, 110–124 (2014).

6. Hue, V., Greathouse, T. K., Cavalié, T., Dobrijevic, M. & Hersant, F. 2D photochemical modeling of Saturn's stratosphere. Part II: Feedback between composition and temperature. *Icarus* **267**, 334–343 (2016). 1512.02724.

7. Brown, L. R. *et al.* Methane line parameters in HITRAN. *Journal of Quantitative Spectroscopy and Radiative Transfer* **82**, 219–238 (2003).

8. Margolis, J. S. Measurement of hydrogen-broadened methane lines in the ν_4 band at 296 and 200K. *Journal of Quantitative Spectroscopy and Radiative Transfer* **50**, 431–441 (1993).

9. Vander Auwera, J., Moazzen-Ahmadi, N. & Flaud, J. Toward an Accurate Database for the 12 μm Region of the Ethane Spectrum. *Astrophysical Journal* **662**, 750–757 (2007).

10. Blass, W. E., Halsey, G. W. & Jennings, D. E. Self- and foreign-gas broadening of ethane lines determined from diode laser measurements at 12 microns. *Journal of Quantitative Spectroscopy and Radiative Transfer* **38**, 183–184 (1987).

11. Halsey, G. W., Hillman, J. J., Nadler, S. & Jennings, D. E. Temperature dependence of the hydrogen-broadening coefficient for the nu 9 fundamental of ethane. *Journal of Quantitative Spectroscopy and Radiative Transfer* **39**, 429–434 (1988).

12. Jacquinet-Husson, N. *et al.* The 2003 edition of the GEISA/IASI spectroscopic database. *Journal of Quantitative Spectroscopy and Radiative Transfer* **95**, 429–467 (2005).

13. Varanasi, P. Intensity and linewidth measurements in the 13.7-micron fundamental bands of (C-12)2H2 and (C-12)(C-13)H2 at planetary atmospheric temperatures. *Journal of Quantitative Spectroscopy and Radiative Transfer* **47**, 263–274 (1992).

14. Kleiner, I. *et al.* NH_3 and PH_3 line parameters: the 2000 HITRAN update and new results. *Journal of Quantitative Spectroscopy and Radiative Transfer* **82**, 293–312 (2003).

15. Levy, A., Lacome, N. & Tarrago, G. Hydrogen- and helium-broadening of phosphine lines. *Journal of Molecular Spectroscopy* **157**, 172–181 (1993).

16. Bouanich, J.-P., Salem, J., Aroui, H., Walrand, J. & Blanquet, G. H_2-broadening coefficients in the ν_2 and ν_4 bands of PH_3. *J. Quant. Spectro. Rad. Trans.* **84**, 195–205 (2004).

17. Salem, J., Bouanich, J.-P., Walrand, J., Aroui, H. & Blanquet, G. Hydrogen line broadening in the ν_2 and ν_4 bands of phosphine at low temperature. *Journal of Molecular Spectroscopy* **228**, 23–30 (2004).

18. Brown, L. R. & Peterson, D. B. An Empirical Expression for Linewidths of Ammonia from Far-Infrared Measurements. *Journal of Molecular Spectroscopy* **168**, 593–606 (1994).

19. Bouanich, J.-P., Blanquet, G., Walrand, J. & Lepère, M. H_2-broadening in the ν_7 band of ethylene by diode-laser spectroscopy. *Journal of Molecular Spectroscopy* **218**, 22–27 (2003).

20. Bouanich, J.-P., Blanquet, G., Walrand, J. & Lepère, M. Hydrogen-broadening coefficients in the ν_7 band of ethylene at low temperature. *Journal of Molecular Spectroscopy* **227**, 172–179 (2004).

21. Jacquinet-Husson, N. *et al.* The 2009 edition of the GEISA spectroscopic database. *Journal of Quantitative Spectroscopy and Radiative Transfer* **112**, 2395–2445 (2011).

22. Waschull, J., Heiner, Y., Sumpf, B. & Kronfeldt, H.-D. Diode Laser Spectroscopy in the 9.8-μm ν $_{14}$Band of Benzene . II. Self-, Air-, and Noble-Gas Broadening Coefficients. *Journal of Molecular Spectroscopy* **190**, 140–149 (1998).
